# Sequential fractionation and isolation of subcellular proteins from tissue or cultured cells

**DOI:** 10.1016/j.mex.2015.11.001

**Published:** 2015-11-07

**Authors:** Sabina Baghirova, Bryan G. Hughes, Michael J. Hendzel, Richard Schulz

**Affiliations:** a Department of Pharmacology, Cardiovascular Research Centre, Mazakowski Alberta Heart Institute, University of Alberta, Edmonton, Alberta, Canada; b Department of Pediatrics, Cardiovascular Research Centre, Mazakowski Alberta Heart Institute, University of Alberta, Edmonton, Alberta, Canada; c Department of Oncology, University of Alberta, Edmonton, Alberta, Canada

**Keywords:** Tissue fractionation, Cell fractionation, Nuclear proteins, Membrane proteins, Subcellular organelles, Sequential lysis

## Abstract

Many types of studies require the localization of a protein to, or isolation of enriched protein from a specific cellular compartment. Many protocols in the literature and from commercially available kits claim to yield pure cellular fractions. However, in our hands, the former often do not work effectively and the latter may be prohibitively expensive if a large number of fractionations are required. Furthermore, the largely proprietary composition of reagents in commercial kits means that the user is not able to make adjustments if, for example, a particular component affects the activity of a protein of interest. The method described here allows the isolation of purified proteins from three cellular fractions: the cytosol, membrane-bound organelles, and the nucleus. It uses gentle buffers with increasing detergent strength that sequentially lyse the cell membrane, organelle membranes and finally the nuclear membrane.

• Quick, simple to replicate or adjust; this method does not require expensive reagents or use of commercial kits
• The protocol can be applied to tissue samples or cultured cells without changing buffer components
• Yields purified fractions of cytosolic, membrane bound and nuclear proteins, with the proper distribution of the appropriate subcellular markers: GAPDH, VDAC, SERCA2 and lamin A/C

## Graphical abstract

**Figure:**
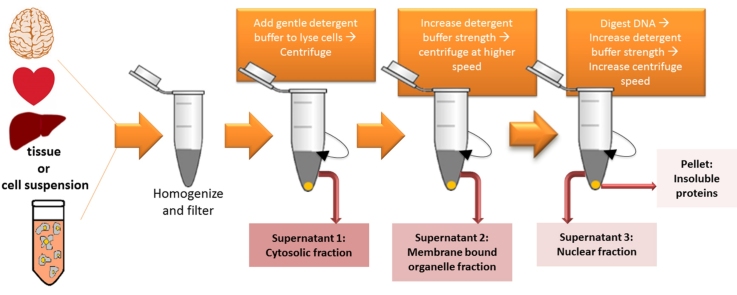

## Method details

This method modifies a previously published protocol based on cultured cells [1], expanding it to allow for the collection of subcellular fractions from fresh tissue. In addition, the buffer compositions (Table 1) were optimized to minimize nuclear protein loss via the addition of 1 M hexylene glycol, which helps to further stabilize the membranes, especially that of the nucleus, and has been previously shown to yield highly enriched nuclear fractions [2]. HEPES is an organic buffer that stabilizes the pH of the solution while NaCl maintains the ionic strength [2].

**Fig. 1:**
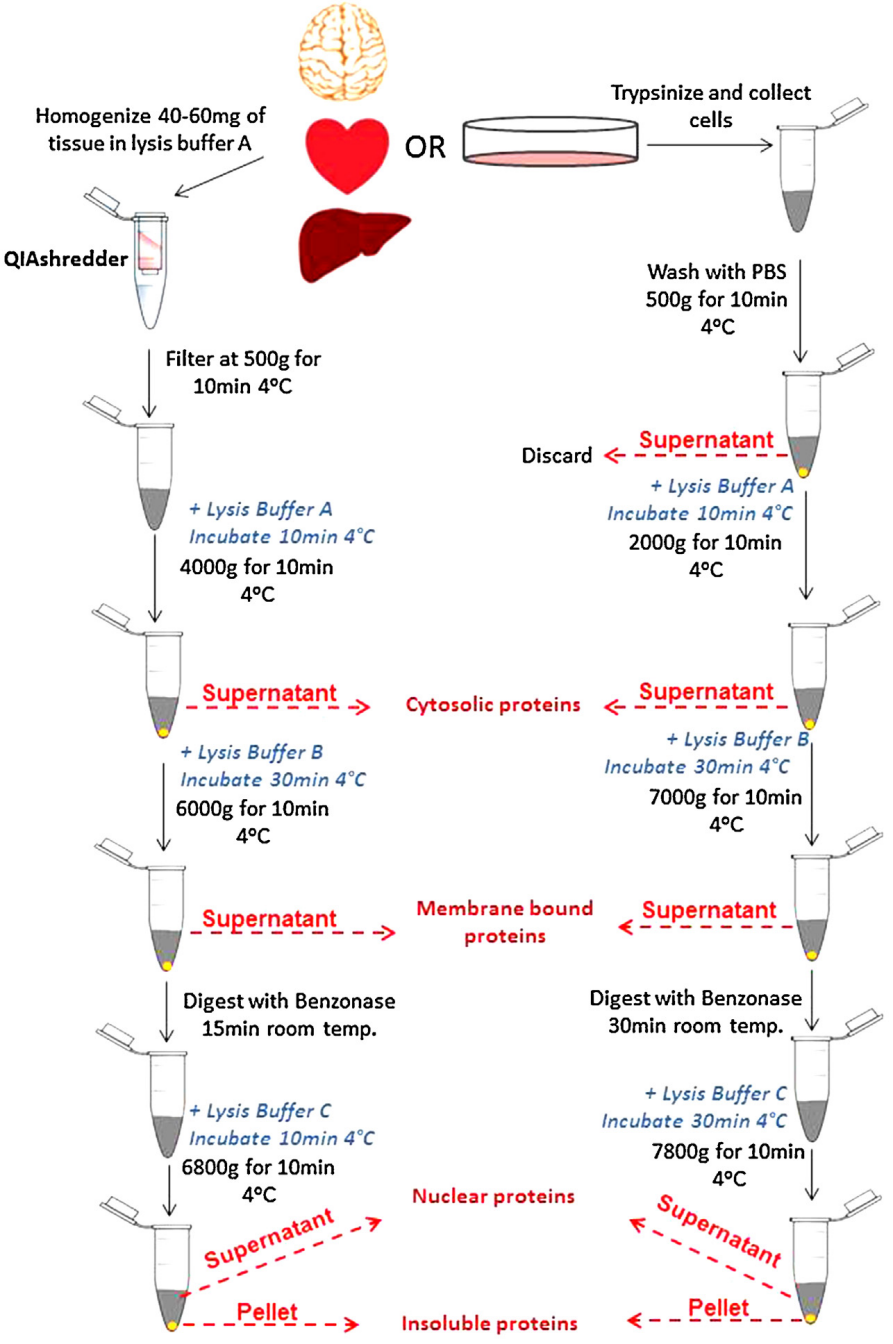
Schematic diagram of the protocol for the fractionation of tissue and cultured cells. The differences between the two methods for tissue and cultured cells are easily identified in this visual diagram.

The basis of this method (Fig. 1) is the sequential lysis of cell membranes by increasing the detergent strength of lysis buffers to obtain proteins from each fraction. Lysis buffer A is meant to release cytosolic proteins and its main component is digitonin. Digitonin is a steroidal saponin that permeabilizes the plasma membrane by binding with cholesterol and other β-hydroxysterols, thereby leading to the formation of pores in the membrane and its subsequent disruption. The advantage of lysing cells with digitonin is that it is unable to disrupt the membranes of cellular organelles, as the cholesterol composition of these membranes is lower [3]. Lysis buffer B releases the proteins from all membrane bound organelles except the nucleus. The main component of this buffer is igepal, which is a non-ionic, non-denaturing detergent chemically equivalent to Nonidet P-40 [4]. It is used at a low concentration to allow permeabilization of the endoplasmic reticulum, Golgi and mitochondria membranes, while keeping the nuclear membrane intact. Lysis buffer C is meant to permeabilize the nuclear membrane and release the nuclear proteins. Among its components, lysis buffer C contains sodium deoxycholate, a mild, non-ionic and non-denaturing biological detergent (a constituent of bile). Sodium dodecyl sulphate is an anionic detergent that is extremely effective in membrane solubilization [5]. The combination of sodium deoxycholate and sodium dodecyl sulphate thus creates an effective nuclear lysis buffer. Benzonase is added prior to the isolation of nuclear fractions and digests DNA and RNA, facilitating the complete release of all nuclear proteins [1].

*Equipment required for this method:*

End-over-end tube rotator
Handheld homogenizer (rotor/stator type)
Qiagen QIAshredder columns (Qiagen, 79656)
Microcentrifuge

*Fractionation protocol for isolated tissue (*Fig. 1*):*

1. Mince fresh, unfrozen tissue into 2–4 mm pieces, wash with 1 mL of ice cold phosphate buffered saline solution (10 mM Na_2_HPO_4_, 2 mM KH_2_PO_4_, 137 mM NaCl, 2.7 mM KCl, pH 7.4).
2. Add 40–60 mg of tissue into a 1.5 mL microtube.
3. Add 500 μL of ice cold lysis buffer A supplemented with 5 μL protease inhibitor cocktail.
4. Disrupt the tissue for 5 s using a hand held tissue homogenizer (rotor/stator type).
5. Transfer the tissue suspension to QIAshredder homogenizer (Qiagen, 79656) and centrifuge at 500 × *g* for 10 min at 4 °C to filter the homogenate.
6. Discard the top filter containing trapped tissue particles, and resuspend the pellet in the filtrate by gently pipetting up and down. Transfer into a new tube.
7. Add 500 μL of ice cold lysis buffer A (1.5 mL if working with brain tissue), and supplement with 5 μL of protease inhibitor cocktail.
8. Incubate the homogenate on an end-over-end rotator for 10 min at 4 °C
9. Centrifuge at 4000 × *g* for 10 min at 4 °C.
10. Collect the supernatant. This fraction contains the cytosolic proteins.
11. Using a 1 mL pipette and tip, resuspend the pellet by gently pipetting up and down in 1 mL of ice cold lysis buffer B supplemented with 10 μL of protease inhibitor cocktail. Incubate for 30 min on an end-over-end rotator at 4 °C.
12. Centrifuge at 6000 × *g* for 10 min at 4 °C.
13. Collect the supernatant. This fraction contains the proteins from membrane-bound organelles (mitochondria, endoplasmic reticulum, Golgi, etc.) except those from the nucleus.
14. Add 500 units of benzonase (Sigma, E1014) to 20 μL of water and combine it with the pellet from step 12.
15. Resuspend the pellet by gently flicking the bottom of the tube and incubate at room temperature for 15 min.
16. Add 500 μL of ice cold lysis buffer C with 5 μL of protease inhibitor cocktail to the benzonase-digested pellet and incubate on an end-over-end rotator for 10 min at 4 °C.
17. Pellet the insoluble material by centrifuging at 6800 × *g* for 10 min at 4 °C.
18. Collect the supernatant. This fraction contains the nuclear proteins.
19. Pellet contains nuclear proteins and protein complexes that resist extraction and typically include active RNA polymerases and regulatory proteins. These can be solubilized with lysis buffer C supplemented with 8 M urea for analysis, or discarded.

*Fractionation protocol for cultured cells (*Fig. 1*):*

The following protocol is optimized for cultured cells grown on a 100 mm diameter dish (55 cm^2^ surface area).

1. Remove culture medium and wash the cells with room temperature phosphate buffered saline solution.
2. Trypsinize the cells by adding 800 μL of 0.25% Trypsin: 0.9 mM EDTA: phenol red solution (Gibco Life Technologies, 25200) and incubating the cells at 37 °C for 2 min or until cells are detached.
3. Add 5 mL of culture medium containing 10% fetal bovine serum to inhibit trypsin activity, and collect the cells.
4. Centrifuge at 500 × *g* for 10 min at 4 °C to pellet the cells.
5. Using a 1 mL pipette and tip, discard the supernatant and resuspend the pellet by pipetting up and down in 500 μL of ice cold PBS.
6. Centrifuge for at 500 × *g* for 10 min at 4 °C to pellet the cells.
7. Discard the supernatant and add 400 μL of ice cold lysis buffer A supplemented with 4 μL of protease inhibitor cocktail.
8. Incubate on end-over-end rotator for 10 min at 4 °C.
9. Centrifuge at 2000 × *g* for 10 min at 4 °C.
10. Collect the supernatant. This fraction contains the cytosolic proteins.
11. Add 400 μL of ice cold lysis buffer B supplemented with 4 μL of protease inhibitor cocktail and resuspend the pellet by vortexing.
12. Incubate on ice (or at 4 °C) for 30 min.
13. Centrifuge at 7000 × *g* for 10 min at 4 °C.
14. Collect the supernatant. This fraction contains the proteins from membrane-bound organelles (mitochondria, endoplasmic reticulum, Golgi, etc.) except those from the nucleus.
15. Add 400 μL of ice cold lysis buffer C containing 7 μL of Benzonase and 4 μL of protease inhibitor cocktail.
16. Incubate on an end-over-end rotator for 30 min at 4 °C to allow complete solubilization of nuclei and digestion of genomic DNA.
17. Centrifuge at 7800 × *g* for 10 min at 4 °C.
18. Collect the supernatant. This fraction contains the nuclear proteins.
19. Pellet contains nuclear proteins and protein complexes that resist extraction and typically include active RNA polymerases and regulatory proteins. These can be solubilized with lysis buffer C supplemented with 8 M urea for analysis, or discarded.

**Fig. 2:**
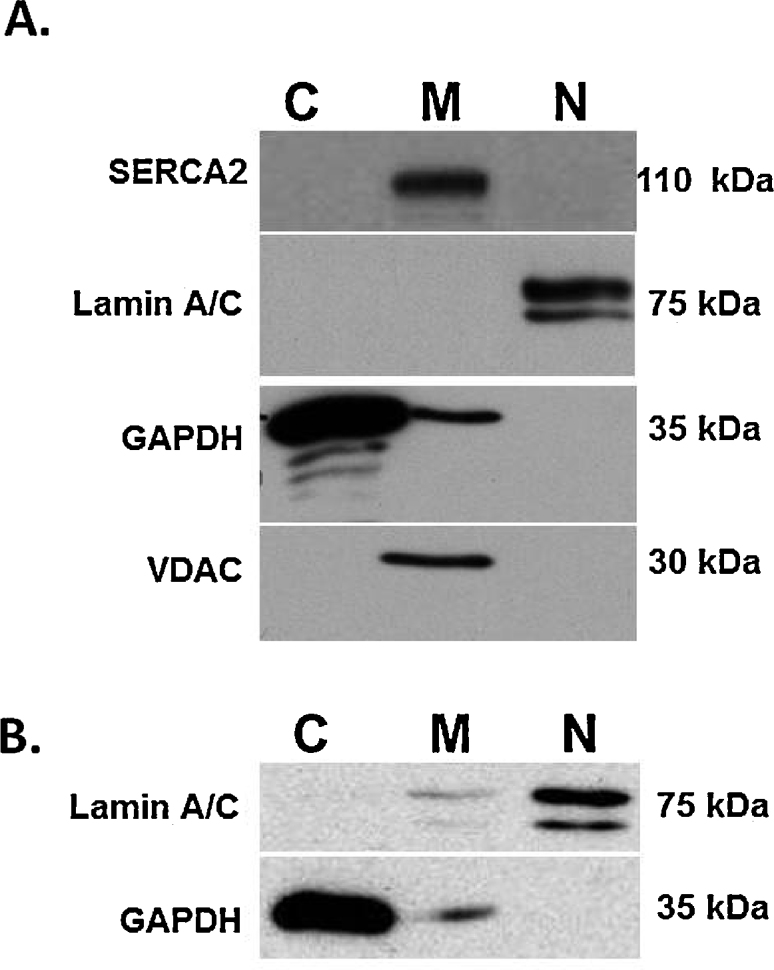
The purity of subcellular fractions from fresh heart tissue isolated from Sprague-Dawley rats (A) or HT1080 human fibrosarcoma cells (B) were assessed by western blotting against specific markers. The cytosolic, membrane bound organelles and nuclear fractions are denoted by C, M and N, respectively. Cytosolic marker: GAPDH; membrane bound organelles markers: SERCA2, VDAC; nuclear marker: Lamin A/C.

Using the technique developed for fractionation of isolated tissue, freshly isolated rat hearts perfused free of blood with Krebs-Henseleit solution at 37 °C using the Langendorff technique [6], were fractionated into cytosolic (C), membrane bound organelle (M) and nuclear (N) fractions. The purity of the fractions was assessed by western blotting against specific markers (Fig. 2). Glyceraldehyde-3-phosphate dehydrogenase (GAPDH, antibody 14C10, Cell Signaling Technology, Beverly, MA, USA) was used as the major cytosolic marker [7]. The sarco/endoplasmic reticulum Ca^2+^-ATPase (SERCA2, antibody ab137020, Abcam, Cambridge, UK) and voltage-dependent anion channel (VDAC, antibody ab15895, Abcam) were used as membrane markers since they are associated with the sarco/endoplasmic reticulum and mitochondria, respectively [8]. Lamin A/C (antibody 2032S, Cell Signaling Technology, Beverly, MA, USA) a major structural protein of the nuclear membrane, was used as a nuclear marker [1].

GAPDH can be found in the mitochondria and small vesicular structures of the cell when exposed to stressors which cause a dynamic subcellular redistribution of GAPDH [7]. Consistent with this, while the bulk of GAPDH is found in the cytosolic fraction, a much smaller amount is present in the membrane fraction. GAPDH is not seen in the nuclear fraction of hearts (Fig. 2A) or human fibrosarcoma HT1080 cells (Fig. 2B), confirming nuclear fraction purity. Both SERCA2 and VDAC were present in the membrane fraction from hearts, but absent from the cytosolic and nuclear fractions (Fig. 2A). Lamin A/C was found exclusively in the nuclear fractions isolated from hearts (Fig. 2A) and primarily in the nuclear fraction isolated from HT1080 cells, with substantially less appearing in the membrane fraction (Fig. 2B). The presence of the nuclear membrane-associated protein lamin A/C mostly in the nuclear fraction indicates that the nuclear membrane is contained in this fraction. These results confirm the purity of the fractions collected from tissue and cells with the protocol presented above.

## Figures and Tables

**Table 1:** Buffers required for the protocol.^a^

**Lysis buffer A**	
NaCl	150 mM
HEPES (pH 7.4)	50 mM
Digitonin (Sigma, D141)	25 μg/mL
Hexylene glycol (Sigma, 112100)	1 M
Protease inhibitor cocktail^b^	1% v:v

**Lysis buffer B**	
NaCl	150 mM
HEPES (pH 7.4)	50 mM
Igepal (Sigma, I7771)	1% v:v
Hexylene glycol	1 M
Protease inhibitor cocktail^b^	1% v:v

**Lysis buffer C**	
NaCl	150 mM
HEPES (pH 7.4)	50 mM
Sodium deoxycholate	0.5% w:v
Sodium dodecyl sulfate	0.1% w:v
Hexylene glycol	1 M
Protease inhibitor cocktail^b^	1% v:v

a Buffers (without protease inhibitor cocktail) can be stored at 4 °C for at least one month.

b Protease inhibitor cocktail (Sigma, P8340) should be added fresh at 1:100 dilution just prior to buffer use.

## References

[1] Holden P., Horton W.A. (2009). Crude subcellular fractionation of cultured mammalian cell lines. BMC Res. Notes.

[2] Sikorskaite S., Rajamäki M.L., Baniulis D., Stanys V., Valkonen J.P.T. (2013). Protocol: optimised methodology for isolation of nuclei from leaves of species in the Solanaceae and Rosaceae families. Plant Methods.

[3] Schulz I. (1990). Permeabilizing cells: some methods and applications for the study of intracellular processes. Methods Enzymol..

[4] Le A.V., Huang D., Blick T., Thompson E.W., Dobrovic A. (2015). An optimised direct lysis method for gene expression studies on low cell numbers. Sci. Rep..

[5] Osteikoetxea X., Sodar B., Nemeth A., Szabo-Taylor K., Paloczi K., Vukman K.V., Tamasi V., Balogh A., Kittel A., Pallinger E., Buzas E.I. (2015). Differential detergent sensitivity of extracellular vesicle subpopulations. Organ. Biomol. Chem..

[6] Skrzypiec-Spring M., Grotthus B., Szelag A., Schulz R. (2007). Isolated heart perfusion according to Langendorff—still viable in the new millennium. J. Pharmacol. Toxicol. Methods.

[7] Tristan C., Shahani N., Sedlak T.W., Sawa A. (2011). The diverse functions of GAPDH: views from different subcellular compartments. Cell. Signal..

[8] Raturi A., Simmen T. (2013). Where the endoplasmic reticulum and the mitochondrion tie the knot: the mitochondria-associated membrane (MAM). Biochim. Biophys. Acta.

